# Opioid-Induced Constipation among a Convenience Sample of Patients with Cancer Pain

**DOI:** 10.3389/fonc.2016.00131

**Published:** 2016-06-08

**Authors:** Karin S. Coyne, Chris Sexton, Robert J. LoCasale, Frederic R. King, Mary Kay Margolis, Sam H. Ahmedzai

**Affiliations:** ^1^Outcomes Research, Evidera, Bethesda, MD, USA; ^2^Medical Evidence and Observational Research in Global Medical Affairs, AstraZeneca, Gaithersburg, MD, USA; ^3^Global Payer Evidence and Pricing in Global Medicines and Development, AstraZeneca, Gaithersburg, MD, USA; ^4^Department of Oncology, University of Sheffield, Sheffield, UK

**Keywords:** opioid, constipation, pain, laxatives, symptoms, cancer

## Abstract

**Background:**

Little is known regarding the burden of opioid-induced constipation (OIC) among patients who suffer from cancer-related pain.

**Methods:**

A prospective longitudinal study was conducted among cancer patients in the United Kingdom (UK), Canada, and Germany, which included medical record data abstraction, Internet-based patient surveys, and physician surveys. Patients on daily opioid therapy (≥30 mg for ≥4 weeks) for treatment of cancer pain with self-reported OIC were recruited. Response to laxatives was defined by classifying participants into categories of laxative use and evaluating the prevalence of inadequate response. Descriptive statistics were used to evaluate outcomes, including the patient assessment of constipation-symptom (PAC-SYM), patient assessment of constipation-quality of life, EuroQOL-5 dimensions, and global assessment of treatment benefit, satisfaction, and willingness to continue.

**Results:**

Recruitment was difficult for this study with only 31 participants completing the baseline survey and meeting criteria for opioid use and OIC (26 UK, 1 Canada, and 4 Germany). Fifty-two percent (*n* = 16) of participants were male, and all were White. Breast (23%, *n* = 7), pancreatic (13%, *n* = 4), and multiple myeloma (13%, *n* = 4) were the most common cancers. Mean duration of chronic pain and opioid use were 2.3 and 1.3 years, respectively. Participants reported having a mean of 4.4 bowel movements/week in the 2 weeks prior to baseline, of which a mean of 0.9 were spontaneous. Most participants (90%, *n* = 28) were using at least 1 lifestyle approach to manage their constipation; 65% (*n* = 20) were taking ≥1 over-the-counter laxative; 19% (*n* = 6) were taking ≥1 prescription laxative; 23% (*n* = 7) reported no laxative use in the prior 2 weeks. Moderate-to-severe constipation symptoms on the PAC-SYM were common, and mean scores on health-related quality of life outcomes were comparable to chronic pain populations.

**Conclusion:**

In this primarily UK sample, there appears to be considerable unmet OIC treatment needs among cancer patients.

## Introduction

At present, there is no consensus definition of opioid-induced constipation (OIC) to guide clinical and epidemiological research or to inform treatment recommendations (1). Depending on the study design and patient population, estimates of the prevalence of OIC range from 15 to 90% based on an analysis of 16 clinical trials and observational studies identified in a systematic review (2) and from 70 to 100% among hospitalized patients (3). The DYONISOS study (DYsfonctiONs Intestinales induiteS par les OpioıdS forts), a cross-sectional observational study conducted among 520 patients with cancer pain in France found that 62% showed a degree of constipation that is problematic according to the patient-reported Knowles–Eccersley–Scott symptom (KESS) score (4). Even more patients (86%) were considered constipated according to physician’s subjective assessment – despite that 85% of these patients reported using laxatives regularly.

Little is known about the decision tree by which cancer patients and their physicians manage OIC. Available management options include lifestyle strategies, such as exercise, increased intake of fiber and fluids, and probiotics, followed by use of laxatives [both over-the-counter (OTC) and prescription treatments] (1). However, these strategies are often ineffective, and patients continue to experience substantial impact on their activities of daily living and overall health-related quality of life (HRQL) (5). Some patients have reported that they would rather endure their pain than continue with the constipating side effects associated with their opioid treatment (6, 7). Thus, OIC can impair adherence to opioid treatment, which can lead to increased morbidity and decreased HRQL in patients with cancer.

In order to better understand OIC among cancer patients, a hybrid, prospective longitudinal study was designed to generate real-world empirical evidence of the patient burden and experience of OIC. Difficulties were encountered when recruiting sites and patients with cancer pain. The recruitment for this patient cohort was truncated after 13 months where 31 patients were enrolled. The objective of this paper is to present the completed baseline data to characterize demographics, clinical characteristics, constipation symptoms, pain management, OIC treatments, rate of inadequate response to laxatives, and the impact of OIC on HRQL in this small convenience sample of patients with cancer pain and OIC.

## Materials and Methods

### Study Design

This analysis focuses on baseline data from a patient survey conducted among patients with cancer pain and is part of an ongoing longitudinal study evaluating clinical characteristics and burden of OIC among patients with cancer and non-cancer pain using a combination of patient surveys, retrospective data abstraction from medical records, and physician surveys (8, 9).

The study received institutional review board approval and was executed in accordance with the Canadian regulation known as the Personal Information Protection and Electronic Documents Act, Health Insurance Portability and Accountability Act of 1996, European Union Data Protection Directive, and Safe Harbor agreement. The protocol (NCT01928953) was also reviewed and endorsed by the Anesthetics Pain Subgroup and the Primary Care Research Network.

The Internet-based patient-reported component includes the baseline survey and eight follow-up Internet surveys conducted over 24 weeks (Weeks 2, 4, 6, 8, 12, 16, 20, and 24). The retrospective chart review was completed by the clinical sites *via* the Internet at baseline and again at the end of the study period (Week 24) for each patient who completed a baseline survey. The Internet-based patient-specific physician survey was completed at baseline and at Week 24 for each patient who completed the baseline survey. Data from the chart review and physician survey are still being collected.

### Study Sample and Data Collection

Additional details about participant recruitment and data collection procedures are described elsewhere in the manuscript summarizing the study methodology and descriptive analysis of baseline study outcomes in the non-cancer pain study sample (8).

Briefly, participants were patients with cancer who were recruited from oncology clinics, primary care clinics, pain management clinics, and clinical research sites affiliated with primary care networks who had chart confirmation of daily opioid therapy lasting ≥4 weeks for the treatment of chronic cancer pain and who self-reported OIC during their screening interview. Clinical site staff identified a preliminary cohort of potentially eligible patients by review of the medical charts at their site. Staff then described the study and the informed consent process to each identified patient. Following completion of the baseline survey, participants were compensated for their time in the form of an electronic gift card in the country’s currency, with values ranging from the equivalent of 15 to 25 USD.

The initial recruitment target for cancer pain patients was 150 patients. However, due to extensive difficulties in recruiting clinical sites to recruit patients and in recruiting cancer pain patients to participate in this OIC burden of illness study, recruitment was truncated after 13 months with only 31 patients enrolled. No sites were activated in the US.

#### Patient Survey

The patient survey included demographic questions regarding gender, age, race, education, marital status, employment status, and insurance type. The survey also included questions about clinical characteristics, including rating of overall health, duration, type, and severity of chronic pain, and duration of opioid use including changes in opioid pain medications to have a bowel movement (BM).

Opioid-induced constipation status was confirmed during the survey by patients meeting one of the following criteria: responding “yes” to question “Since starting the opioid pain medication, have you been experiencing constipation or worsening of current constipation? Yes/No”; OR reporting fewer than three BMs per week, OR reporting laxative use if more than three BM per week, OR report at least one symptom of OIC on the patient assessment of constipation-symptom (PAC-SYM) in the past 2 weeks. Participants’ answered questions about their history of constipation, including questions about the duration of constipation symptoms and health-care seeking in relation to constipation symptoms, as well as questions about which therapies the health-care provider (HCP) suggested for constipation. Information about participants’ bowel symptoms, including BM frequency, spontaneity, and preferences, were collected in addition to which therapies they used to relieve their constipation. Management options included lifestyle approaches (probiotics, increased fluids, increased exercise, and natural diet change), OTC therapies (fiber supplements, stool softeners, and laxatives), and prescription laxatives.

The PAC-SYM questionnaire (10) was designed to assess the severity of patient-reported symptoms over the previous 2 weeks. The 12-item measure assesses three subscales: stool, rectal, and abdominal symptoms. Each item is rated on a five-point Likert scale, ranging from 0 to 4, where a higher score indicates greater symptom severity. The PAC-SYM has been widely used in OIC medication treatment studies and has performed as expected in relation to clinical outcomes (11–14). The PAC-SYM items and additional constipation symptoms, as well as the frequency of discomfort and degree of bother of each symptom, were collected at each survey completion.

Finally, participants also completed patient-reported questionnaires assessing condition-specific and general HRQL, including

1. The patient assessment of constipation-quality of life (PAC-QOL): a 28-item measure designed to capture four QOL domains: physical discomfort, psychological discomfort, worries and concerns, and satisfaction (which was not measured in this study) with a recall period of 2 weeks. Similar to the PAC-SYM, participants are asked to rate themselves over the previous 2 weeks on a five-point Likert scale ranging from 0 to 4, where higher scores indicate worse QOL.
2. The EuroQOL-5 dimensions (EQ-5D) is a five-item health state descriptive system where full health is 1 and 0 is equivalent to death (EQ-5D index), and a visual analog scale (EQ-5D VAS, range 0–100 from worst to best imaginable health state). There is extensive evidence supporting the reliability and validity of the EQ-5D (15), and it has performed well in a range of therapeutic areas, including OIC (16). The EQ-5D was completed by participants on-site in a paper-based format, and the data were entered into the Internet-based data capture system. Scoring was based on developer guidelines and weighted by country (15).
3. The global assessment of treatment benefit, satisfaction, and willingness (BSW) to continue consists of three single-item measures that are designed to capture patients’ perception of the effect of their treatment in terms of benefits, satisfaction, and willingness to continue treatment (17). The BSW has demonstrated evidence of construct validity in patients with overactive bladder and has been used in urology treatment studies (17, 18) and the non-cancer pain sample (8).

### Statistical Analysis

Analyses were performed using SAS version 9.4 (SAS Institute, Cary, NC, USA) following a statistical analysis plan approved prior to receipt of the locked and clean dataset. The data were analyzed as observed, without imputation for missing responses. Descriptive statistics were used to evaluate outcomes. For continuous variables, the mean and SD were described and rounded to one decimal for all data. For categorical variables, the number and percent distribution by category were described.

Participants who completed the baseline survey were further evaluated for presence of OIC in order to confirm study eligibility, which included the following criteria.

Participants who responded “Yes” to the baseline question: “Since starting opioid pain medication, have you been experiencing constipation or worsening of current constipation? Yes/No”; OR participants who reported laxative use if the number of BMs in the past 2 weeks is ≥3/week; OR participants who reported <3 BMs/week in the past 2 weeks were eligible, regardless of whether they reported laxative use; OR participants who did not report laxative use but reported at least one symptom of OIC at least “moderate” or more often on the PAC-SYM screening questions in the past 2 weeks (i.e., incomplete BM, BM too hard, straining during BM, or sensation of false alarm).

Participants were classified into groups based on their use of laxatives, which included the following: stool softeners [e.g., docusate sodium (Colace^®^, Ducolax^®^ stool softener), mineral oil], osmotics [e.g., polyethylene glycol 3350 (MiraLAX^®^, Dulcolax^®^ balance)], stimulants [bisacodyl (Ducolax^®^ laxative), senna (Senokot^®^, Exlax^®^)], salines [magnesium hydrochloride (Milk of Magnesia^®^, Citroma^®^)], rectal options (suppositories, enemas), and prescription therapies [polyethylene glycol 3350, lactulose (Constulose^®^), Lubiprostone (Amitiza^®^), and methylnaltrexone bromide (Relistor^®^)].

Sufficient laxative use was defined as use of at least one laxative ≥4 times over the last 2 weeks; insufficient laxative use was defined as use of at least one laxative <4 times but ≥1 time over the last 2 weeks, and non-laxative use was defined as no reported laxative use over the last 2 weeks. These categories were created to quantify laxative exposure in relation to other outcomes and do not imply clinical sufficiency.

## Results

### Demographic and Clinical Characteristics

All 31 (100%) participants completing the baseline survey met criteria for opioid use and OIC. Eighty-four percent (*N* = 26) were from the United Kingdom (UK), 3% (*N* = 1) was from Canada, and 13% (*N* = 4) were from Germany. Just over half (*n* = 16, 52%) were male, and all were White (Table 1). Most were married (*n* = 28) and unable to work due to disability or handicap (*n* = 11) or retired (*n* = 16).

**Table 1:** **Participant demographics**.

	Baseline (*N* = 31)^a^
Gender (*N*, % male)	16 (51.6)
Age (mean, SD years)	60.7 (10.0)
Race (*N*, % White/Caucasian)	31 (100.0)
Marital status	
Married	28 (90.3%)
Divorced	1 (3.2%)
Widowed	2 (6.5%)
Employment status	
Employed, full-time	2 (6.5%)
Employed, part-time	1 (3.2%)
Unable to work due to disability or handicap	11 (35.5%)
Not currently working for pay (e.g., full-time homemaker)	1 (3.2%)
Retired	16 (51.6%)

*^a^Eligible population defined as all participants recruited into the study and who have baseline survey data, are on opioids per protocol, and met criteria for opioid-induced constipation*.

Most participants (*n* = 24) reported discussing their OIC with their HCP during an office visit (Table 2). The same proportion reported that their doctor explained that constipation was a potential side effect of opioid treatment. Among participants who discussed their constipation symptoms with their HCP in the prior month, 85% reported their HCP offered helpful advice for treatment of their symptoms.

**Table 2:** **Patient-reported baseline clinical characteristics and pain**.

	Baseline (*N* = 31)
Discussed OIC with HCP during office visit (*N*, %) (*N* = 27 who had office visit)	24 (88.9)
Health rating (*N*, %)	
Excellent	0 (0.0)
Very good	1 (3.2)
Good	4 (12.9)
Fair	13 (41.9)
Poor	13 (41.9)
Type of chronic pain (*N*, %)^a^	
Pain related to cancer	30 (96.8)
Back pain	15 (48.4)
Joint pain	10 (32.3)
Pain syndrome	10 (32.3)
Neuralgia	3 (9.7)
Fibromyalgia	2 (6.5)
Rheumatoid arthritis	2 (6.5)
Headache or migraine	1 (3.2)
Other^b^	2 (6.5)
Pain severity	
Average pain last 24 h (mean, SD)	5.2 (2.5)
Average pain last 7 days (mean, SD)	5.2 (2.6)
How much does the constipation interfere with your ability of opioid medication to control pain? (*N*, %)	*N* = 28
No interference; pain adequately managed	5 (17.9)
Little interference, pain mostly managed	11 (39.3)
Moderate interference, pain moderately managed	11 (39.3)
Complete interference with adequate pain management; pain not at all managed	1 (3.6)
In the past 7 days, did you change how you used your opioid pain medication(s), so that you could have BMs? (*n*, % yes)	3 (9.7)
If yes: how change (*N*, %)	*N* = 3
No longer take my pain medication	0 (0.0)
Reduced how much of my pain medication I use	2 (66.7)
Temporarily interrupted the use of pain medication	0 (0.0)
Switched to a different pain medication	0 (0.0)
Other	1 (33.3)

*^a^This category is not mutually exclusive, and participants were able to report more than one type of chronic pain*.

*^b^Other includes liver metastases (*N* = 1) and leg/ankle cramp (*N* = 1)*.

*BM, bowel movement; HCP, health-care provider; OIC, opioid-induced constipation*.

Most patients reported that their health was fair (*n* = 13) or poor (*n* = 13). The mean (±SD) duration of pain was 2.3 ± 3.6 years, and the mean (±SD) duration of opioid medication use was 1.3 ± 1.3 years. In addition to pain related to their cancer, patients commonly reported back pain (*n* = 15), joint pain (*n* = 10), and pain syndrome (*n* = 10). Many participants (*n* = 12) reported that their constipation moderately or completely interfered with the ability of their opioid medication to control pain.

Nearly, half (*n* = 15) were on more than one opioid, with morphine being the most frequently prescribed opioid (*n* = 13) followed by oxycodone (*n* = 12). The mean morphine equivalent dose (MED) at baseline was 183.3 ± 185 mg, and five patients had a MED of ≥200 mg/day. Nine patients discontinued use of their opioids over the course of the study and mean MED at week 24 for remaining patients was 251.3 ± 313.4 mg.

### Constipation and Bowel Symptoms

Participants reported 4.4 ± 2.5 BMs on average per week, with 0.9 ± 1.5 BMs being spontaneous. Eighty-seven percent of participants (*n* = 26) reported wanting to have a BM at least once a day or more. Thirty participants reported at least 1 of the 12 PAC-SYM symptoms as moderate or greater in intensity (Table 3). Not only did these symptoms occur with moderate or greater severity but also the symptoms occurred frequently in at least 25% of each BM for most patients: discomfort in abdomen (*n* = 27), pain in abdomen (*n* = 24), bloating in abdomen (*n* = 23), painful BM (*n* = 21), incomplete BM (*n* = 22), BMs too hard (*n* = 19), straining/squeezing to pass BM (*n* = 22), and feeling like had to pass BM but could not (*n* = 19).

**Table 3:** **PAC-SYM items and subscales and additional at least moderate constipation symptoms**.

	Baseline (*N* = 31)
Any symptom ≥ moderate present, *N* (%)	30 (96.8)
Discomfort abdomen, *N* (%)	23 (74.2)
Pain in abdomen, *N* (%)	20 (64.5)
Bloating in abdomen, *N* (%)	24 (77.4)
Stomach cramps, *N* (%)	14 (45.2)
Painful BM, *N* (%)	22 (71.0)
Rectal burning during or after BM, *N* (%)	12 (38.7)
Rectal bleeding/tearing during or after BM, *N* (%)	7 (22.6)
Incomplete BM, *N* (%)	16 (51.6)
BMs too hard, *N* (%)	18 (58.1)
BMs too small, *N* (%)	12 (38.7)
Straining/squeezing to pass BM, *N* (%)	17 (54.8)
Feeling like had to pass BM but could not, *N* (%)	16 (51.6)
Additional symptoms	
Nausea, *N* (%)	4 (12.9)
Vomiting, *N* (%)	2 (6.5)
Flatulence, *N* (%)	20 (64.5)
Gastroesophageal reflux, *N* (%)	9 (29.0)
Headache or migraine, *N* (%)	2 (6.5)

*BM, bowel movement; OIC, opioid-induced constipation; PAC-SYM, patient assessment of constipation symptoms*.

### Laxative Use

The most frequently utilized OIC management options in the past 2 weeks were natural or behavior therapies, including dietary changes, increased fluids or exercise, probiotics, and fiber supplements (*n* = 28; Table 4). At least 1 OTC laxative, such as stool softeners, osmotic laxatives, saline laxatives, or rectal options, was used by 20 participants and 6 used 1 or more prescription agents. Over half of participants (*n* = 16) used a combination of at least one natural or behavioral therapy and one OTC therapy.

**Table 4:** **Summary of natural/behavioral therapies and laxative utilization for management of OIC**.

	Baseline (*N* = 31)
Use within each category (past 2 weeks)	
Natural/behavioral approaches^a^ (*N*, %)	
≥1	28 (90.3)
≥2	17 (54.8)
≥3	9 (29.0)
OTC laxatives^b^ (*N*, %)	
≥1	20 (64.5)
≥2	8 (25.8)
≥3	2 (6.5)
Prescription agents^c^ (*N*, %)	
≥1	6 (19.4)
≥2	2 (6.5)
≥3	1 (3.2)
Natural/behavioral approaches and laxative combinations (*N*, %)	
Natural/behavioral approaches and OTC laxative	16 (51.6)
≥1 Natural/behavioral therapies only	6 (19.4)
≥1 OTC only	2 (6.5)
Natural/behavioral approaches, OTC laxative, and prescription agent	2 (6.5)
Natural/behavioral approaches and prescription agent	4 (12.9)
No natural/behavioral approach or laxative use	1 (3.2)

*^a^Includes probiotics, natural dietary changes, increased fluids, increased exercise, and fiber supplements*.

*^b^Includes stool softeners, osmotic laxatives, saline laxatives, rectal options, and other OTC products*.

*^c^Includes prescription osmotic laxatives, lactulose, Amitiza^®^, Relistor^®^, and other prescription agents*.

*OTC, over-the-counter*.

Twenty-two participants met the study criteria for sufficient laxative use, defined as use of at least one laxative four or more times over the past 2 weeks, two had insufficient laxative use, and seven reported no laxative use at all in the prior 2 weeks.

### Health-Related Quality of Life and Perceived Treatment Benefit

Patient-reported outcomes on the PAC-QOL, EQ-5D, and BSW reflected the considerable disease burden of this population. The mean (±SD) PAC-QOL physical discomfort, psychosocial discomfort, and worries and concerns domain scores were 1.7 ± 1.0, 1.2 ± 1.1, 1.9 ± 1.1, respectively (Table 5). The mean (±SD) EQ-5D index score was 0.54 ± 0.28, and the mean (±SD) EQ-5D VAS score was 54.4 ± 16.3 (Table 5).

**Table 5:** **PAC-QOL and EQ-5D**.

	Baseline (*N* = 31)
PAC-QOL (mean, SD)^a^	
Physical discomfort	1.7 (1.0)
Psychosocial discomfort	1.2 (1.1)
Worries and concerns	1.9 (1.1)
EQ-5D index^b^	*N* = 30
Mean (SD)	0.54 (0.28)
EQ-5D VAS^c^	*N* = 30
Mean (SD)	54.4 (16.3)

*^a^Scores range from 0 to 4 with lower scores indicating better quality of life*.

*^b^Country-specific weights were used to calculate scores (15, 19). EQ-5D Index scores range from 0 (death) to 1 (full health)*.

*^c^The EQ-5D VAS ranges from 0 (“worst imaginable health state”) to 100 (“best imaginable health state”)*.

*EQ-5D, EuroQol-5 dimension; VAS, visual analog scale*.

Twenty-three participants reported on the BSW questionnaire that they had some benefit from their treatment for constipation, with the majority of those who reported benefit characterizing it as “little” (*n* = 13, 57%; Table 6). Nonetheless, all but one participant reported being willing to continue their current treatment for constipation.

**Table 6:** **Benefit, satisfaction, and willingness to continue treatment**.

	Baseline (*N* = 31)
Have you had any benefit from your constipation treatment? *N* (%)	
No	8 (25.8)
Yes	23 (74.2)
*If yes, how much benefit? N (%)*	
Little benefit	13 (56.5)
Much benefit	10 (43.5)
Taking all things into account, are you satisfied with your constipation treatment? *N* (%)^a^	*N* = 30
No	10 (33.3)
*If no, how dissatisfied? N (%)*	
A little dissatisfied	6 (60.0)
Very dissatisfied	4 (40.0)
Yes	20 (66.7)
*If yes, how satisfied? N (%)*	
A little satisfied	8 (40.0)
Very satisfied	12 (60.0)
Would you be willing to continue constipation treatment with this medication? *N* (%)	
No	1 (3.2)
*If no, how unwilling? N (%)*	
A little unwilling	1 (100.0)
Very unwilling	0 (0.0)
Yes	30 (96.8)
*If yes, how willing? N (%)*	
A little bit willing	6 (20.0)
Very willing	24 (80.0)
Missing	0 (0.0)

*^a^Due to a problem with the programing of the survey, this question was not presented to one participant at baseline. The data are missing*.

## Discussion

This study was conducted to generate real-world empirical evidence to inform medical understanding of the OIC burden and its management among patients with cancer pain. Participants commonly reported a range of constipation symptoms as assessed by the PAC-SYM. These findings contribute to the understanding of the OIC experience by signaling some consistencies and a few notable inconsistencies when compared to our previously reported results conducted among the portion of the study sample with chronic non-cancer pain (20) despite the notable sample size discrepancy and lack of US presence in the cohort.

The prevalence of sufficient laxative use reported in this descriptive sample of cancer patients with OIC (*n* = 22, 71%) was higher than what was found among patients with OIC and non-cancer pain (*n* = 234, 48%). This may be due to the higher rates of discussion regarding OIC between the physicians and patients in the cancer cohort (*n* = 24, 77%) than non-cancer cohort (*n* = 309, 63%). Participants with cancer pain also reported a greater number of BMs per week on average (4.4) as compared to those with non-cancer pain (3.7), though fewer of these were spontaneous (cancer pain sample, 0.9; non-cancer pain sample 1.4), and the majority of patients in both samples (cancer pain sample, 84%; non-cancer pain sample, 83%) reported wanting to have a BM at least once a day or more. Interestingly, the proportion of participants reporting PAC-SYM constipation symptoms at least moderate or more often tended to be higher among participants in the cancer pain cohort compared to those with non-cancer pain. Patients in the non-cancer cohort reported significantly longer durations of pain (mean ± SD years: non-cancer, 9.8 ± 8.9; cancer, 2.3 ± 3.6) and opioid use (mean ± SD years: non-cancer, 6.4 ± 6.3; cancer, 1.3 ± 1.3), which may have contributed to this difference in results as the non-cancer pain patients have been coping with OIC for much longer and have developed a new “normal” with OIC.

Despite this considerable symptom burden, patients with cancer pain and OIC characterized their overall HRQL as slightly better than those with non-cancer chronic pain (EQ-5D index 0.54 cancer pain patients; 0.49 non-cancer pain; EQ-5D VAS cancer pain patients 54.4; non-cancer pain patients 50.4).

In terms of constipation-specific quality of life, participants with cancer pain in this study had PAC-QOL scores that were similar to those reported among patients with constipation and cancer pain in the DYONISOS study (4). As expected, these values were considerably higher, reflecting greater impairment in HRQL due to constipation, than those found in patients with cancer pain who do not suffer from constipation (4).

Taken together, these findings suggest that patients with cancer pain commonly experience burdensome constipation symptoms despite being more likely to proactively treat their OIC using sufficient laxatives as compared to patients with non-cancer pain. Previous publications evaluating laxative management in ambulatory cancer patients also suggest substantial unmet treatment needs (1). Wirz et al. (21) found that 53% of patients discontinued their laxative prescription in a prospective, open-label investigation of polyethylene glycol (PEG), sodium picosulphate, and lactulose. Importantly, Wirz et al. (21) found that those who stopped taking laxatives had significantly lower opioid doses – which could suggest that patients may compromise their management of pain in order to avoid symptoms of constipation and the negative side effects resulting from available laxative treatment options as supported by Bell et al. (22). Indeed, 43% (*n* = 12) of cancer pain patients in this study reported that OIC at least moderately interfered with their pain management.

Although most patients with cancer pain and OIC are willing to continue their current laxative treatment regimen, there is a need for viable treatment alternatives with less burdensome side effect profiles and better efficacy. Novel peripherally acting μ-opioid antagonists may be useful in reversing opioid agonist-induced actions, including bowel dysfunction (23).

There are a number of limitations to this study. First, as noted above, recruitment was prematurely terminated due to the difficulty encountered when recruiting sites and cancer patients with OIC. Reasons for recruitment difficulties were thought to be due to cancer pain patients being burdened with research and other treatment considerations, which reduced the likelihood and interest to participate in non-interventional, observational research.

Thus, the small sample size (*n* = 31) is limited in its generalizability and precision with respect to point estimates on the characteristics reported. The cohort is composed of patients with a range of different cancer diagnoses, levels of disease severity, and other health problems leaving this a descriptive analysis of the burden of OIC in a small number of cancer pain patients. Second, bowel habits in this study were not able to be compared to those prior to opioid use, so patients with a long-standing history of constipation could not be differentiated from those who had OIC. Third, the use of an Internet-based survey may have biased the study sample to those with higher levels of education and socioeconomic status. Finally, most of the study questions used a 1-week (7-day) recall period, which may have resulted in recall bias in reporting symptoms that vary from day to day. These limitations aside, the study does highlight the burden of OIC among patients with cancer pain.

## Conclusion

Although most patients with cancer pain and OIC in this predominantly UK sample were willing to continue their current laxative treatment regimen, there appears to be a need for viable treatment alternatives with better efficacy. Further research on OIC burden and management of the condition is needed. To do so, challenges in recruiting sites and patients need to be elucidated and resolved, so that future research design for studies related to OIC in cancer pain can generate point estimates with acceptable precision. Increased awareness of OIC and its persistence among this already burdened patient cohort may be needed among HCPs.

## Author Contributions

All authors participated in the conceptualization and writing of this paper, have seen and approved the final manuscript, meet the criteria for authorship, and approved its submission to Frontiers in Oncology.

## Conflict of Interest Statement

SA is affiliated with the University of Sheffield. FK and RL are employed by AstraZeneca. KC is employed by Evidera, which provides consulting and other research services to pharmaceutical, device, government and non-government organizations. MM and CS were employed by Evidera at the time the study was completed and during the writing of this manuscript. As Evidera employees, they work with a variety of companies and organizations and are expressly prohibited from receiving any payment or honoraria directly from these organizations for services rendered. MM, CS, and KC have received consultancy fees from AstraZeneca.
